# Smartphone-Based Approach-Avoidance Bias Modification for Alcohol Stimuli: Proof-of-Concept Study

**DOI:** 10.2196/89635

**Published:** 2026-09-10

**Authors:** Matthis Morgenstern, Benjamin Pietsch, Hilmar Zech, Anya Pedersen, Reiner Hanewinkel

**Affiliations:** 1 Institute for Therapy and Health Research, Harmsstr. 2, Kiel, Schleswig-Holstein, 24114, Germany, 49 431-570-2935, 49 431-570-2929; 2 Technische Universität Dresden, Dresden, Saxony, Germany; 3 Christian-Albrechts-Universität zu Kiel, Kiel, Schleswig-Holstein, Germany

**Keywords:** approach-avoidance task, cognitive bias modification, mobile health, automatic action tendencies, alcohol-related disorders, mHealth

## Abstract

**Background:**

Targeting automatic approach tendencies toward alcohol-related stimuli has been shown to significantly reduce relapse rates in patients with alcohol use disorder (AUD) when used as an add-on to inpatient treatment. Traditional approach-avoidance task (AAT) training requires a clinical setting and a stationary device.

**Objective:**

A mobile app was tested that allowed the training to be conducted outside a clinical environment while capturing actual motion parameters, such as acceleration.

**Methods:**

Three proof-of-concept studies assessed the reliability and validity of the mobile app version of the AAT. Study 1 tested technical stability and measurement reliability in 26 university students across 6 home-based sessions. Study 2 examined approach-avoidance tendencies in 15 inpatients with AUD. Study 3 tested 28 soccer fans to determine the modifiability of alcohol-related biases after 3 or 6 training sessions.

**Results:**

The app demonstrated high technical stability and measurement reliability in study 1. In study 2, patients showed faster reaction times (RTs) when pushing away alcohol-related images (avoidance tendency) and greater acceleration when pulling them toward themselves (approach tendency). In study 3, participants also exhibited an RT-based alcohol avoidance bias and a small acceleration-based alcohol approach bias. Training effects were observed for RTs after the test, indicating an increase in alcohol avoidance. An increase in acceleration-based alcohol avoidance was only found in participants with risky alcohol consumption.

**Conclusions:**

The mobile app version of the AAT appears to produce reliable measures and might have the potential to effectively modify alcohol-related automatic tendencies. The current study serves as a proof of concept, so all results should be treated as exploratory. Future randomized controlled trials are needed to evaluate the app’s potential to change actual alcohol behavior in nonclinical populations.

## Introduction

In 2019, alcohol consumption was associated with 2.6 million deaths and 116 million disability-adjusted life years lost [1]. It is a leading global health concern due to its strong association with chronic diseases, including several forms of cancer. Epidemiological evidence indicates a dose-response relationship between alcohol intake and cancer risk: the more alcohol consumed, the higher the probability of developing cancer [2]. Exceeding weekly alcohol intake thresholds of 200 g significantly reduces life expectancy by 1 to 2 years, with reductions of up to 5 years at consumption levels exceeding 350 g per week [2].

In Germany, alcohol consumption remains widespread despite modest declines over recent decades. In 2017, the average per capita consumption among individuals aged ≥15 years was 10.5 L of pure alcohol per year [3]. This places Germany among high-consumption nations, in contrast to countries such as Norway or Greece, where per capita consumption is 6 to 6.5 L of pure alcohol. Evidence-based alcohol control policies, such as taxation, restricted availability, advertising bans, and public education, are associated with reductions in population-level alcohol intake and related adverse health effects [4,5]. However, Germany continues to display a relatively permissive approach toward alcohol as a “cultural good,” contributing to persistently high consumption levels [4].

Findings in health and clinical psychology highlight that addictive behaviors, including alcohol misuse, are strongly influenced by automatic cognitive processes that operate largely outside conscious awareness [6]. According to dual-process models of behavior, human actions result from the interplay between fast, implicit (automatic) and slow, explicit (controlled) cognitive systems [7,8]. In addiction, this balance is disrupted, with excessive impulsive responses toward substance-related cues and diminished cognitive control [9,10]. Consequently, individuals may continue substance use despite consciously recognizing its negative consequences, as automatic approach biases drive craving and relapse [11].

The approach-avoidance task (AAT) has been developed to measure automatic approach and avoidance tendencies toward specific stimuli [12]. In this paradigm, participants push or pull a joystick in response to images of various orientations, thereby simulating avoidance or approach movements. Reaction time (RT) differences between push and pull trials indicate an underlying bias, that is, faster push or pull reactions dependent on image content. The AAT has been validated across multiple domains, such as fear of spiders [12] and social anxiety [13], and later adapted for addiction research; alcohol-dependent individuals typically exhibit stronger approach biases toward alcohol-related stimuli than toward neutral cues [14], while similar biases have been observed for cannabis-related [15], nicotine-related [16], and heroin-related [17] stimuli.

The AAT paradigm has been successfully extended into therapeutic applications. Such AAT-based neurocognitive training, also known as cognitive bias modification, aims to retrain automatic action tendencies by repeatedly pairing alcohol-related stimuli with avoidance responses. Clinical studies have demonstrated that such training, when added to standard inpatient treatment for alcohol dependence, significantly reduces relapse rates for up to 12 months after discharge [18]. One limitation of these existing studies is, however, that they rely on laboratory-based setups, requiring special response joysticks. This increases participant burden, restricts accessibility, and ultimately limits scalability.

To address these limitations, recent developments have focused on adapting AAT-based training for mobile platforms, enabling real-world use and repeated self-administered interventions. Mobile AAT app versions using touch screen gestures [19,20] or motion sensors [21] have demonstrated technical feasibility and user engagement. A mobile neurocognitive training app may therefore represent a scalable, low-threshold tool for measuring and modifying automatic approach biases in both clinical and preventive contexts, potentially broadening access to evidence-based behavioral interventions beyond traditional treatment settings.

The aim of the present feasibility study was to determine whether automatic approach tendencies toward alcohol stimuli can be reliably measured “at home” using an app-based AAT and whether systematic avoidance training with alcohol-related stimuli can modify these tendencies. Specifically, the study sought to assess the reliability of the app’s measurements, the influence of image content on automatic action tendencies in both clinical and nonclinical samples, changes of action tendencies with repeated training, and the usability of the app.

## Methods

### Ethical Considerations

This study was reviewed and approved by the ethics committee of the Medical Faculty of Kiel University (AZ 487/23), which raised no ethical or legal objections to the research protocol. Written informed consent was obtained from all participants prior to their inclusion in the study. Participants were informed that their participation was voluntary and that they had the right to withdraw at any time without penalty. All data collection and processing were conducted in accordance with the General Data Protection Regulation. Participants were compensated with credit points (study 1) or gift vouchers (study 3).

### The Mobile AAT App (Used in All Studies)

Approach-avoidance tendencies were measured with a smartphone-based AAT app (Zech et al [21]), which was delivered through the Android OS. During the use of the mobile AAT app, participants are presented with stimuli on the smartphone screen and are asked to respond to the stimuli by pulling the smartphone toward themselves (approach movement) or pushing the smartphone away from themselves (avoidance movement). During these movements, the smartphone’s accelerometer tracks rotation- and gravity-corrected acceleration from which RTs and subsequently RT-based approach and avoidance tendencies can be calculated. In addition, the smartphone measures peak acceleration, from which acceleration-based approach and avoidance tendencies can be derived by comparing the force that is used to pull or push the smartphone.

### Experimental Procedure

At the beginning, a short video illustrated the correct pull and push movements as well as the appropriate arm and smartphone positioning (landscape format). A relevant feature task was applied; specifically, participants were instructed to respond to the content of the presented pictures (alcohol vs neutral) by either pulling or pushing the smartphone. A total of 20 alcohol and 20 neutral stimuli, pretested for valence and arousal, were presented in a random order. The initial task assignment (“pull alcohol or push neutral” vs “push alcohol or pull neutral”) was also randomized across participants. After 40 stimulus presentations, midway through the task, the participants took a 2-minute break, during which a new instruction page was presented. For the second half, participants were instructed to perform the opposite movement pattern (eg, pulling alcohol pictures if they had pushed them during the first half).

### Study 1: Reliability Test in Psychology Students

Twenty-six psychology students from Kiel University participated in the study, earning course credit for their involvement. Participants attended an initial on-site introduction to the app and experiment and then completed the remaining sessions independently at home. Students were allowed to use their own smartphones or, if they did not have an Android phone, were provided with a rental smartphone (either a Motorola G52 or a Samsung Galaxy A04s). Each participant completed 6 sessions in the course of 1 week, with 80 measurements per session. RT (measured in ms) and maximum acceleration of the smartphone (measured in m/s²) were recorded.

### Study 2: Automatic Response Tendencies in Patients With Alcohol Use Disorder

Overall, 15 inpatients (87% male) undergoing treatment at the Freudenholm-Ruhleben clinic participated in the study. Each participant completed the alcohol AAT individually on a Samsung Galaxy A04s. In addition to the task, participants completed the 4-item short Obsessive Compulsive Drinking Scale to assess craving. RT and maximum acceleration were also recorded.

### Study 3: Automatic Response Tendencies and Training Effects in Soccer Fans With Overweight

In total, 28 participants with overweight from 3 soccer clubs were enrolled via the Soccer Fans in Training program [22]. Initial training instructions, and the before and after measurements of automatic tendencies were provided on-site, and the training sessions were completed at home. The prebias and postbias measurements were identical to the other 2 studies. The training sessions consisted of 120 measurements, divided into 3 phases of 40 measurements each. The 120 measurements were based on 10 alcohol and 10 neutral stimuli, that is, each stimulus was presented 6 times in total. Order of presentation was random within each phase. Training instruction was to always push pictures with alcohol content. Participants were allowed to use their own smartphones or, if they did not have an Android phone, were provided with a rental phone (either a Motorola G52 or a Samsung Galaxy A04s). There were 2 training conditions (3 vs 6 training sessions), which were cluster randomized at the club level. Participants from 2 clubs (n=17) received 3 training sessions, while participants from 1 club (n=11) were assigned to the 6-session condition. Alcohol Use Disorders Identification Test-Consumption (AUDIT-C) scores were collected to classify risky alcohol consumption (score ≥4).

### Statistical Analysis

Statistical analyses were performed using Stata software (version 18; StataCorp). Internal consistency was evaluated using Cronbach α, and split-half reliability was assessed using random splits. To analyze continuous behavioral outcomes (RT and maximum acceleration), linear mixed-effects models were fitted using the mixed command with maximum likelihood estimation. Models were specified with fixed effects for all main effects and 2-way, 3-way, and 4-way interaction terms between stimulus type (alcohol vs neutral), movement direction (approach vs avoid), number of training sessions (eg, treated as a continuous variable or factor variable), and baseline AUDIT-C score. To account for repeated measures within individuals, random intercepts for participants were included in the random-effects structure. For illustration purposes, D-scores were calculated [23], which control for differences in individual response time and force of movement. For RT, D-scores were calculated as follows: ([*mean alcohol avoid–mean alcohol approach*]*/pooled alcohol SD*)*–*([*mean neutral avoid–mean neutral approach*]*/pooled neutral SD*)*.* For maximum acceleration, D-scores were calculated as follows: ([*mean alcohol approach–mean alcohol avoid*]*/pooled alcohol SD*)*–*([*mean neutral approach–mean neutral avoid]/pooled neutral SD*). Thus, for both measures, positive D-scores indicate an alcohol approach tendency, whereas negative D-scores indicate an alcohol avoidance tendency.

The following responses were removed before the analyses in all 3 studies: (1) RTs <200 ms, (2) RTs >2000 ms, (3) incorrect responses, and (4) nonresponses. Linear mixed-effects models handle missing values under the missing-at-random assumption; thus, all available observations were retained in the primary analyses without imputation. Marginal means derived from the interaction terms were calculated using the margins command.

## Results

### Study 1: Reliability Test

Of the 26 students (n=20, 77% female; aged 18‐36 years), 23 (88%) completed all 6 sessions. Invalid values (<100 ms or >2000 ms or nonresponse) averaged 2.7% across sessions, with a variation of 0.5% to 9.2% (Table 1). Correct movements (“pull” if instruction was “pull” and “push” if instruction was “push”) were performed in 85% to 92% of trials. Mean RTs and accelerations remained stable across sessions. Split-half reliabilities ranged from *r*=0.80 to 0.98 for RT and *r*=0.94 to 0.97 for maximum acceleration. Within-session internal consistency was high for both alcohol-related and neutral stimuli, with Cronbach α ranging from 0.81 to 0.98, depending on movement type (pull vs push) and outcome (Table 2). RT and maximum acceleration correlated negatively with *r*=−0.09 across all sessions, with the smallest correlation found in session 1 (*r*=−0.09) and the highest in session 6 (*r*=−0.18).

**Table 1.** Measurement statistics per session.

Sessions	Total, n	Valid values (%)	Correct responses (%)	Average response time (ms)	Average maximum acceleration (m/s^2^)
1	26	99.5	89.9	609	11.1
2	24	98.8	91.7	592	9.9
3	24	97.5	89.5	597	10.6
4	23	90.8	85.0	588	10.4
5	23	98.5	90.4	593	10.9
6	23	96.5	89.3	598	10.3

**Table 2.** Cronbach α values by outcome (reaction time vs acceleration), stimulus category (alcohol vs neutral), and movement type (push vs pull)^a^.

Outcome, stimulation category, and movement type	Cronbach α
Reaction time	
Alcohol	0.94
Push	0.88
Pull	0.86
Neutral	0.89
Push	0.81
Pull	0.90
Acceleration	
Alcohol	0.94
Push	0.95
Pull	0.98
Neutral	0.95
Push	0.97
Pull	0.98

a On the basis of data from the first session, with 80 measurements per person per outcome.

### Study 2: Validity Test

For RT, the analysis revealed a significant 2-way interaction between movement type and stimulus type (*z* score=−3.84; *P*<.001). Inpatients receiving treatment for alcohol use disorder (AUD) were faster to push away alcohol-related images (557 ms) than to pull them toward themselves (576 ms), whereas neutral images elicited faster pull responses (599 ms) than push responses (646 ms). This pattern indicates an avoidance bias for alcohol pictures (D-score=−0.52). Craving moderated this effect (3-way interaction *z* score=−3.71; *P*<.001), such that participants with higher craving exhibited stronger avoidance (D-score=−0.70) than those with lower craving (D-score=−0.32).

**Figure 1.**
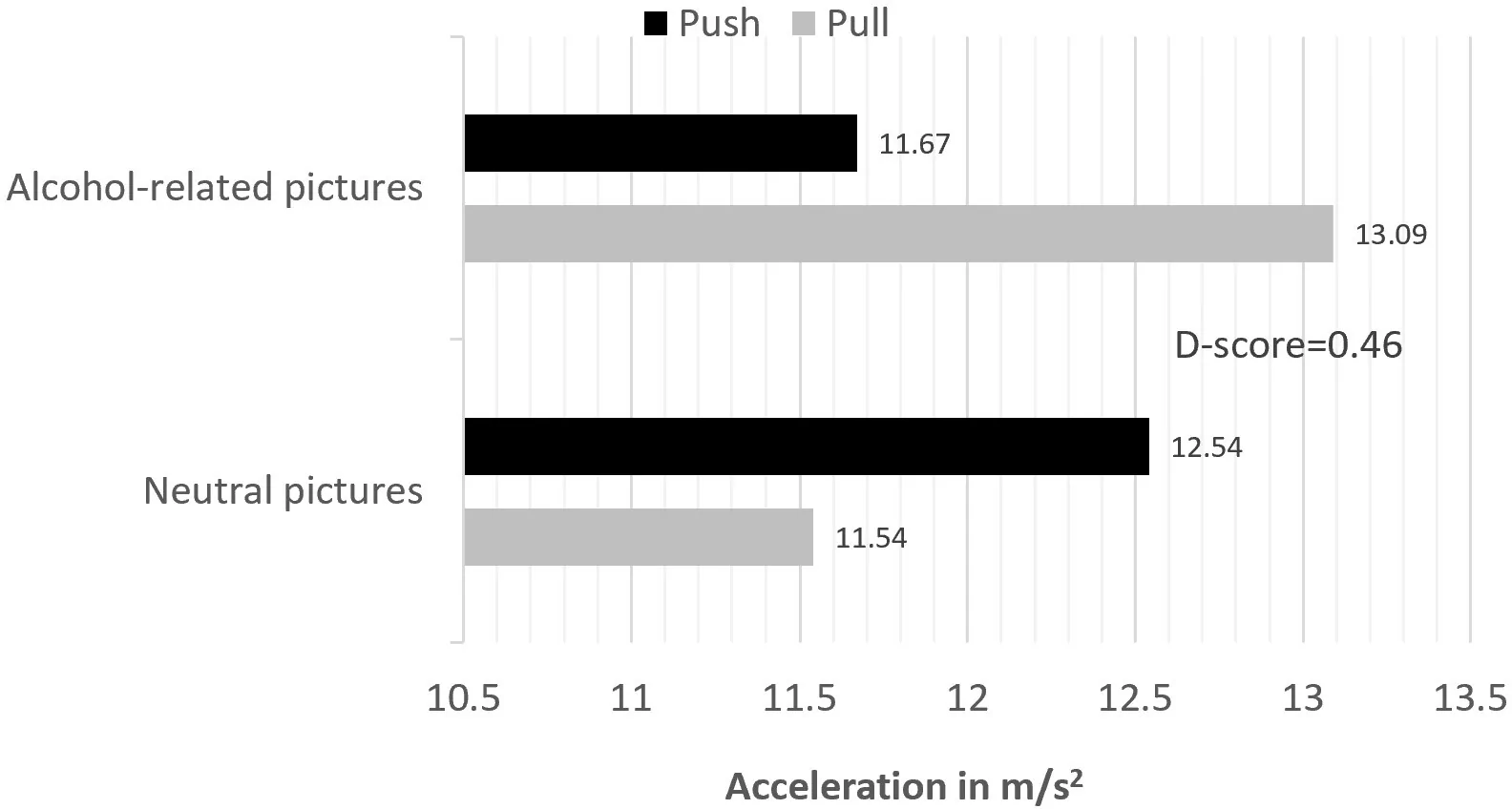
Mean maximum acceleration of the smartphone among inpatients, by stimulus category and movement direction. A positive D-score indicates a tendency to approach alcohol.

The analysis of maximum acceleration indicated an approach bias for alcohol pictures (Figure 1). Inpatients moved the smartphone with higher force if they were instructed to pull alcohol-related pictures (13.1 m/s²) than to push them (11.7 m/s²), while it was the opposite for neutral stimuli (11.5 m/s² for pull vs 12.5 m/s² for push; *z* score=−5.14; *P*<.001). Craving did not influence acceleration.

### Study 3: Changes in Reaction Tendencies Over Time

The pretest measurements showed an avoidance tendency in RT for alcohol-related images (D-score=−0.22; Table 3). After the app training, this avoidance tendency had increased (D-score=–0.78), with a significant 3-way interaction among movement, stimulus, and time (*z* score=2.43; *P*=.02). Maximum acceleration did not differ significantly at pretest (D-score=0.09) and did not change over time (D-score after the test=0.07). However, there was a significant 4-way interaction between movement, stimulus, time, and AUDIT-C score (*z* score=−2.08; *P*=.04), indicating that participants without risky consumption changed from a small alcohol avoidance tendency (D-score=−0.08) to an alcohol approach tendency (D-score=0.41), while soccer fans with risky consumption changed from an alcohol approach tendency (D-score=0.22) to an alcohol avoidance tendency (D-score=−0.18). There were also significant 4-way interactions between movement, stimulus, time, and dose for both outcomes (Table 3). For RTs, this interaction revealed that avoidance learning was stronger if the training dose was higher (*z* score=3.45; *P*=.001). For maximum acceleration, app training led to an increase in acceleration over time across all movement and stimulus types in the 6-session group, while the 3-session group showed a reduction in acceleration. The increase in acceleration in the 6-session group was pronounced for “pull alcohol” and “push neutral,” resulting in an approach tendency (D-score=0.37) after the test.

**Table 3.** Reaction tendencies of soccer fans before and after the approach-avoidance task app training, overall and stratified by the Alcohol Use Disorders Identification Test-Consumption (AUDIT-C) score and training dose (3 vs 6 training sessions)^a^.

	Before the test					After the test					Interaction, *P* value
	Push alc^b^	Pull alc	Push neut^c^	Pull neut	D-score^d^^,^^e^	Push alc	Pull alc	Push neut	Pull neut	D-score^d^^,^^e^	
Reaction time (ms)											
Total (n=28)	583.8	599.1	648.0	619.5	−0.22	572.6	612.0	638.1	583.8	−0.78	.01^f^
AUDIT-C											.65^g^
<4 (n=12)	588.2	593.7	631.4	624.7	−0.01	580.1	608.2	632.9	610.8	−0.46	
>3 (n=16)	580.5	603.4	661.8	615.5	−0.38	566.9	614.9	642.2	562.8	−1.01	
Sessions											.001^h^
3 (n=17)	585.1	624.8	674.7	617.0	−0.50	575.3	608.3	645.5	588.4	−0.82	
6 (n=11)	581.7	559.5	605.5	624.5	0.20	568.3	617.8	626.3	576.4	−0.71	
Maximum acceleration (m/s^2^)											
Total (n=28)	14.43	14.95	13.85	13.91	0.09	14.23	14.49	14.32	13.79	0.07	.67^f^
AUDIT-C											.04^g^
<4 (n=12)	17.54	17.55	16.59	16.14	−0.08	16.08	16.82	17.06	15.96	0.41	
≥4 (n=16)	13.01	12.09	11.78	12.24	0.22	12.85	12.75	12.24	12.16	−0.18	
Sessions											.007^h^
3 (n=17)	14.70	16.10	14.28	15.01	0.13	14.16	14.32	13.46	13.69	−0.12	
6 (n=11)	14.00	13.19	13.20	12.17	0.03	14.34	14.76	15.69	13.93	0.37	

a Positive D-scores indicate an alcohol approach tendency, and negative D-scores indicate an alcohol avoidance tendency. *P* values were based on linear mixed models with random intercepts for individuals (on average, 73 measures per person per time).

b alc: alcohol-related pictures.

c neut: neutral pictures.

d Double difference D-score “RT”: ([Push_alc–Pull_alc]/pooled_SD_alc)–([Push_neut–Pull_neut]/pooled_SD_neut).

e Double difference D-score “Acceleration”: ([Pull_alc–Push_alc]/pooled_SD_alc)–([Pull_neut–Push_neut]/pooled_SD_neut).

f 3-way-interaction: Push_Pull×Stimulus_content×Time.

g 4-way interaction: Push_Pull×Stimulus_content×AUDIT-C×Time.

h 4-way interaction: Push_Pull×Stimulus_content×3vs6sessions×Time.

## Discussion

The current pilot study investigated the feasibility of using a smartphone-based AAT (alcohol AAT) to measure and modify automatic response tendencies toward alcohol-related stimuli. The study demonstrated several key findings. First, the smartphone’s motion sensors provided reliable and stable measurements across sessions, supporting the feasibility of the app-based assessment. Second, the app detected systematic reaction tendencies in clinical patients, with faster push-away responses to alcohol-related images (indicative of avoidance) and higher acceleration when pulling alcohol-related images toward themselves (indicative of approach tendencies). Third, similar systematic tendencies were observed in a nonclinical sample of soccer fans with overweight and risky alcohol consumption. Fourth, app training increased avoidance tendencies toward alcohol-related stimuli, particularly among participants with risky baseline alcohol consumption as measured by the AUDIT-C.

The rationale for this investigation is grounded in the recognition that many addictive behaviors, including alcohol use, are strongly influenced by automatic cognitive processes, which operate largely outside conscious control [6]. These automatic processes can drive consumption despite awareness of negative health consequences, a phenomenon that has been conceptualized using dual-process models of behavior [7-9]. Previous clinical applications of the AAT paradigm have primarily focused on patients with alcohol dependence, where neurocognitive training was used as an adjunct to standard therapy. In these studies, patients were trained to avoid alcohol-related stimuli, which resulted in measurable changes in approach-avoidance tendencies and reductions in relapse rates over 12 months [18]. However, these interventions were limited to stationary computer setups using joysticks, restricting applicability outside clinical or laboratory environments. Mobile or app-based implementations have been largely limited to touch screen or keyboard simulations that fail to capture genuine approach and avoidance movements [19,20]. The current study addresses this limitation by using a mobile app capable of measuring actual arm movements, thereby extending the technical and conceptual scope of the AAT paradigm [21]. Despite ongoing debates regarding the precise underlying mechanisms of approach and avoidance tendencies, there is a consensus that approach motivations prompt individuals to reduce physical distance to a stimulus, whereas avoidance motivations drive them to increase it [24,25]. In contrast, RT, the sole measurement in previous studies, is defined as the time between picture onset to the first movement of the smartphone; some type of effect anticipation is needed to use RT as an implicit measure of approach or avoidance. It might be conceptually clearer to assess both RT and acceleration, as many AAT studies intermix these 2 response dimensions by defining RT as the time between picture onset and movement completion.

Empirically, it remains unclear what this specific mobile acceleration task app measures. Response force has been shown to be closely related to motivation strength in humans [26] and is traditionally used to measure approach-avoidance motivation in animals [27-29]. The results of the present study suggest that it might indeed be related to approach or avoidance reactions, as are results of other studies [21,30]. However, the small correlation between the two measures (RT and acceleration) suggests that they may not simply represent 2 different ways of measuring the same construct. The validity test (study 2) with inpatients in treatment for AUD did not find an alcohol approach bias with the RT measure, which contrasts with many previous studies [18,31,32]. This can be a measurement artifact but could also be a reflection of active coping, motivational state, and/or task implementation (eg, using a relevant vs irrelevant feature task). Other studies also found an alcohol avoidance bias in early treatment inpatients using a relevant feature task [33], which was explained by interference between conscious motivation to stop drinking and automatic processes. This interference might be smaller with the acceleration measure, although such an assumption needs further empirical validation.

Significant limitations of this proof-of-concept study are, of course, the small sample sizes and the highly selective convenience samples. No control groups were recruited, and no sham training was included. Some of the results were unexpected and need to be treated with high caution, for example, faster RTs if instruction was “push alcohol.” Furthermore, while RT-based tendencies have a long history on approach-avoidance tendencies, acceleration-based tendencies are a relatively novel measure; therefore, these results should be interpreted as exploratory and potentially unstable. Another limitation relates to the assignment of training dose in study 3, which occurred at the club rather than individual level, potentially introducing clustering and selection effects. Most importantly, the present proof-of-concept study did not assess changes in actual drinking behavior; therefore, the app cannot be seen as clinically relevant or effective.

While the pilot study does not yet impact clinical practice, the smartphone-based approach offers a promising way for preventive interventions targeting larger populations. In nonclinical samples, the app could potentially be used for primary prevention to maintain low-risk drinking behaviors and abstinence or for selective prevention and early intervention to reduce risky alcohol consumption.

In conclusion, the present study provides initial exploratory evidence that a smartphone-based AAT can reliably measure and modify alcohol-related automatic response tendencies. By enabling scalable, home-based delivery of AAT training, this approach has the potential to complement traditional prevention and intervention strategies and extend their reach to individuals at risk of developing alcohol-related problems. Future research should focus on randomized controlled trials to evaluate whether modifications in automatic approach-avoidance tendencies translate into sustained behavioral change and reduced health risks in diverse populations.

## Abbreviations

AAT: approach-avoidance task
AUD: alcohol use disorder
AUDIT-C: Alcohol Use Disorders Identification Test-Consumption
RT: reaction time
